# Effects of Rapamycin Combined with Low Dose Prednisone in Patients with Chronic Immune Thrombocytopenia

**DOI:** 10.1155/2013/548085

**Published:** 2013-12-02

**Authors:** Jiaming Li, Zhaoyue Wang, Lan Dai, Lijuan Cao, Jian Su, Mingqing Zhu, Ziqiang Yu, Xia Bai, Changgeng Ruan

**Affiliations:** Key Lab of Thrombosis and Hemostasis of Ministry of Health, Jiangsu Institute of Hematology, The First Affiliated Hospital of Soochow University, 188 Shizi Street, Suzhou 215006, China

## Abstract

We conducted this randomized trial to investigate the efficacy and safety of rapamycin treatment in adults with chronic immune thrombocytopenia (ITP). Eighty-eight patients were separated into the control (cyclosporine A plus prednisone) and experimental (rapamycin plus prednisone) groups. The CD4^+^CD25^+^CD127^low^ regulatory T (Treg) cells level, Foxp3 mRNA expression, and the relevant cytokines levels were measured before and after treatment. The overall response (OR) was similar in both groups (experimental group versus control group: 58% versus 62%, *P* = 0.70). However, sustained response (SR) was more pronounced in the experimental group than in the control group (68% versus 39%, *P* < 0.05). Both groups showed similar incidence of adverse events (7% versus 11%, *P* = 0.51). As expected, the low pretreatment baseline level of Treg cells was seen in all patients (*P* < 0.001); however, the experimental group experienced a significant rise in Treg cell level, and there was a strong correlation between the levels of Treg cells and TGF-beta after the treatment. In addition, the upregulation maintained a stable level during the follow-up phase. Thus, rapamycin plus low dose prednisone could provide a new promising option for therapy of ITP.

## 1. Introduction 

Immune thrombocytopenia (ITP) is an acquired autoimmune disease characterized by an autoantibody-mediated destruction and impaired platelet production. Recently, it has become evident that the impairment of Treg cells may contribute to the development of ITP [1–4]. They play a critical role in the maintenance of peripheral tolerance by suppressing self-reactive lymphocytes. Once these regulating cells are impaired, patients have activated autoreactive T cells against platelet and imbalanced cytokine production, which accelerate the destruction of platelets [5–7]. Given the defective function or low cell numbers of Tregs in patients with ITP, expansion of the functional Treg cells represents an interesting therapeutic approach. In addition, some clinical studies have demonstrated that the effective treatments for ITP can improve the Treg cells level after the platelet count is recovered [8–11]. Although the exact mechanism is not fully understood, these results suggest a promising possibility that Treg cells could be a potential biomarker to therapies in the future.

Rapamycin, as an immunosuppressant, has been used safely and effectively to treat renal transplant rejection since 1999 [12]. By inhibiting the intracellular kinase mTOR, rapamycin can selectively expand the functional Treg cells [13–17]. These expanded Treg cells suppress proliferation of T cells in vitro and prevent allograft rejection in vivo [18]. Subsequently, a large number of research reported that rapamycin spared and promoted growth of functional Treg cells in the field of transplantation immunology and autoimmune diseases [19–24]. Until now, due to the safety and efficacy of rapamycin in clinical trials, it is under more intensive investigation for the treatment of various immune-mediated disorders, including type 1 diabetic, systemic lupus erythematosus and rheumatoid arthritis [25, 26]. However, the effect of rapamycin on human Treg cells and the mechanisms responsible for the rapamycin-mediated Treg cells expansion in ITP patients were not explored. Since the decreased number and function of the Treg cells was involved in the mechanisms in ITP [27, 28], we performed this prospective clinical trial using rapamycin with low dose prednisone in the treatment of patients with chronic ITP, particularly, through determining the alternation of the Treg cells as well as long term clinical outcomes.

## 2. Subjects and Methods

### 2.1. Patients

This observational study began in 2011 and is ongoing. Ethical approval for the study was obtained from the Jiangsu Institute of Hematology. Eighty-eight patients were enrolled in our study, signed the informed consents before this study, and were randomly assigned to the control or experimental group. Patients' inclusion criteria included a diagnosis of ITP according to the guidelines of the American Society of Hematology and the duration was more than 12 months. The platelet count was less than 30 × 10^9^/L or 50 × 10^9^/L if patients displayed the hemorrhagic manifestations. Patents had been off ITP medications (except for prednisone less than 20 mg/day). Exclusion criteria included HIV, HCV serology, or HBsAg positivity, positive pregnancy test, other diseases known to be associated with ITP, such as human immunodeficiency or lymphoproliferative disorders, thyroid or liver disease, definite systemic lupus erythematosus, and definite antiphospholipid syndrome; patients were excluded from the study if they had an abnormal clinical picture aside from their symptoms of ITP or were unlikely to comply with the protocol.  Table 1 summarizes patients' main characteristics at baseline (Table 1). There were 61 females and 27 males, with a median age of 35 years (range 13–65 years). The median time from diagnosis to treatment was 3 years (range 2–6 years) and the median platelet count before treatment was 22 × 10^9^/L (range 11–35 × 10^9^/L). All patients needed chronic treatment to maintain a safe number of platelet count and had been resistant to one or more different therapeutic regimens (Steroids, Rituximab, Danazol, Azathioprine, and Vincristine), while no one was splenectomized.

### 2.2. Administration and Criteria for Response

Patients in the experimental group received rapamycin (6 mg on the first day, then 2 mg per day oral administration) plus low dose of prednisone (10–20 mg per day) and patents in the control group received cyclosporine A (3 mg/kg orally twice daily) with prednisone (10–20 mg/day), respectively. For patients who responded well to the treatment, the treatment was continued for 3 to 6 months after their platelet count increased to normal range, while for unresponsive patients, after 3 months treatment, an alternative regime or splenectomy was provided.

The criteria for response to treatments were as follows. (1) Complete response (CR): the platelet count greater than 100 × 10^9^/L; (2) partial response (PR): the platelet count 30–100 × 10^9^/L and also doubled baseline count after treatment; (3) no response (NR): the platelet count less than 30 × 10^9^/L; (4) overall response (OR): a partial or complete response; (5) sustained response (SR): the platelet count was more than 50 × 10^9^/L during the follow-up phase. (6) nonsustained response (non-SR): patients requiring another treatment to manage ITP due to their low platelet counts. Any patient who lost their best response but did not need further treatment was not considered as failure.

### 2.3. Sample Collection

Peripheral blood samples were collected at the following points: (1) before the treatment in all patients (PB1); (2) after the treatment in patients who obtained the best response; (3) during the follow-up period in patients with OR. The blood samples without anticoagulant were centrifuged and the serum was stored at −40°C. Peripheral blood mononuclear cells (PBMC) were obtained by density gradient centrifugation on Ficoll-Hypaque (density (*d*) = 1.077 g/L, Amersham Biosciences) for 25 min at 1500 g, washed three times in phosphate-buffered solution (PBS) before adding 1ml Trizol reagent (Invitrogen, USA), and then stored at −40°C.

### 2.4. Flow Cytometry 

The flow cytometry of T lymphocytes was performed for each patient. Anti-CD4 conjugated to chlorophyll protein (PerCP), anti-CD8 conjugated to phycoerythrin (PE), and anti-CD127 conjugated to phycoerythrin (PE), anti-CD25 conjugated to fluorescein isothiocyanate (FITC) and isotype-matched control antibodies were obtained from Beckman Coulter. The Treg cells were determined by staining with surface markers CD4-PerCP, CD127-PE, and CD25-FITC. Cells were analyzed by a FC500 flow cytometry (Beckman Coulter, USA). Total events of 50 000 were gated based on forward (FSC) and side-scatter (SSC) characteristics and dot plots for Treg cells were gated on CD4^+^cells. Treg cells were defined as CD25^+^CD127^intensity/dim^ coexpression and expressed as a percentage of total CD4^+^ T population.

### 2.5. Quantitative RT-PCR Assay

Total RNA was extracted from PBMC with Trizol reagent, and cDNA synthesis was performed using the SuperScript II reverse transcriptase kit (Invitrogen) with random hexamers. The reaction was in a 20 *μ*L at 70°C for 5 minutes, 37°C for 1 hour, and 95°C for 5 minutes. For amplification of gene-specific products, equal concentration of cDNA 1 *μ*L from various samples, gene-specific primers 0.2 *μ*M, and Taq DNA polymerase 0.5 U (Invitrogen) were included in 25 *μ*L PCR reaction, and ABL gene was used as the internal control. The probe sequences for Foxp3 and ABL were as follows, respectively: FAM-5-tcc aga gaa gca gcg gac act caa tg-3-TAMRA and FAM-5-tgc ttc tga tgg caa gct cta cgt ctc ct-3-TAMRA (Applied Biosystems, USA). After 5 minutes at 95°C, the amplification was carried out by 45 cycles at 95°C for 10s and 60°C for 60s. Threshold cycle values were obtained for each sample depending on its gene content. Mean duplicate measurements were normalized and expressed as a ratio of FoxP3 mRNA copies/ABL mRNA copies. This assay was performed in triplicate for all patients and controls.

### 2.6. Enzyme-Linked Immunosorbent Assay

The serum levels of interleukin IL-2, IL-4, and IL-10 were determined using commercially available enzyme-linked immunosorbent assay from Invitrogen. For the plasma TGF-*β* assay, platelet-poor plasma was prepared in order to minimise the contribution of platelet degranulation. The assay was performed in triplicate and the concentrations were calculated from a standard curve according to the manufacturer's protocol.

### 2.7. Cells Purification

PBMC were isolated from 7 patients (5 in responsive group, 2 in unresponsive group) before and after the rapamycin treatment and 5 healthy controls using Ficoll density gradient centrifugation method. The Treg cells were purified by staining with surface marker CD4-PC5, CD127-PE, and CD25-FITC. At the same time, CD4^+^ T cells were purified from PBMCs by negative selection using monoclonal antibodies directed against CD8, CD14, CD16, CD19, and CD56 (Beckman Coulter) and combined with sheep anti-mouse IgG (Beckman Coulter). CD8^+^ T cells were purified from PBMCs also by negative selection using monoclonal antibodies against CD4, CD14, CD16, CD19, and CD56. The purity of sorted populations was routinely >95%.

### 2.8. Suppression Assay

The purified CD4^+^ T cells or CD8^+^ T cells (2 × 10^4^/well) labeled with CFSE (Molecular, USA) were stimulated with plate-bound anti-CD3 (2 *μ*g/mL) and anti-CD28 (2 *μ*g/mL) (Peprotech, USA), respectively, as described elsewhere [29]. The freshly isolated Treg cells of these patients at a suppressor to responder cell ratio of 1 : 1 were cocultured in the 200 *μ*L culture medium (RPMI-1640 supplemented with 10% FBS, 4100 U/mL penicillin, 100 *μ*g/mL streptomycin) containing IL-2 (200 IU/mL), at 37°C, 5% CO_2_ in 96-well round bottomed plates. Cell division was assessed at day 5 after stimulation by FACS analysis of CFSE dilution.

### 2.9. Statistical Analysis

Continuous variables are expressed as mean± SDs. Paired *t* test measurement of repeated multiple analysis was performed to the level of Treg cells and TGF-beta between the two groups at the same phase, or during different phases of treatment and followup. Analysis of one-way ANOVA was used to determine difference between patients with sustained response and patients with nonsustained response. Analysis of potential predictive factors was performed by the use of logistic regression, including age, sex, duration, previous treatments, and baseline platelet count. All *P* values were two-tailed. Results were considered statistically significant when the two-sided *P* value was less than 0.05. All data were analysed with SPSS version 17.0 (SPSS Inc., Chicago, IL, USA).

## 3. Results

### 3.1. Treatment Phase

#### 3.1.1. Efficacy and Safety

The OR rates were similar in the rapamycin group and the cyclosporine A group (58% versus 62%, *P* = 0.7): the CR rates were 15/43 (35%) in the rapamycin group and 17/45 (38%) in the cyclosporine A group; the PR rates were 10/43 (23%) and 11/45 (24%), respectively. The median onset time was 1.96 months (1–3 months) in the rapamycin group versus 1.71 months (1–3 months) in the cyclosporine A group; the median time to CR/PR was 3.28 months (2–4 months) versus 2.96 months (2–4 months) and the median treatment time was 8.48 months (7–10 months) versus 8.04 months (6–10 months).

All patients completed the therapeutic program as scheduled. Rapamycin or cyclosporine A was well tolerated although some patients experienced mild gastrointestinal reaction such as nausea, vomiting, and diarrhea. Two patients in the rapamycin group and 2 in the cyclosporine A group developed secondary hypertension; 1 patient in the rapamycin group and 2 in the cyclosporine A group suffered from the respiratory infection; 1 patient in the cyclosporine A group experienced hyperlipemia. These abnormalities came to normaly without any other therapy during treatment phase.

#### 3.1.2. Immunologic Assessment

**Figure 1:**
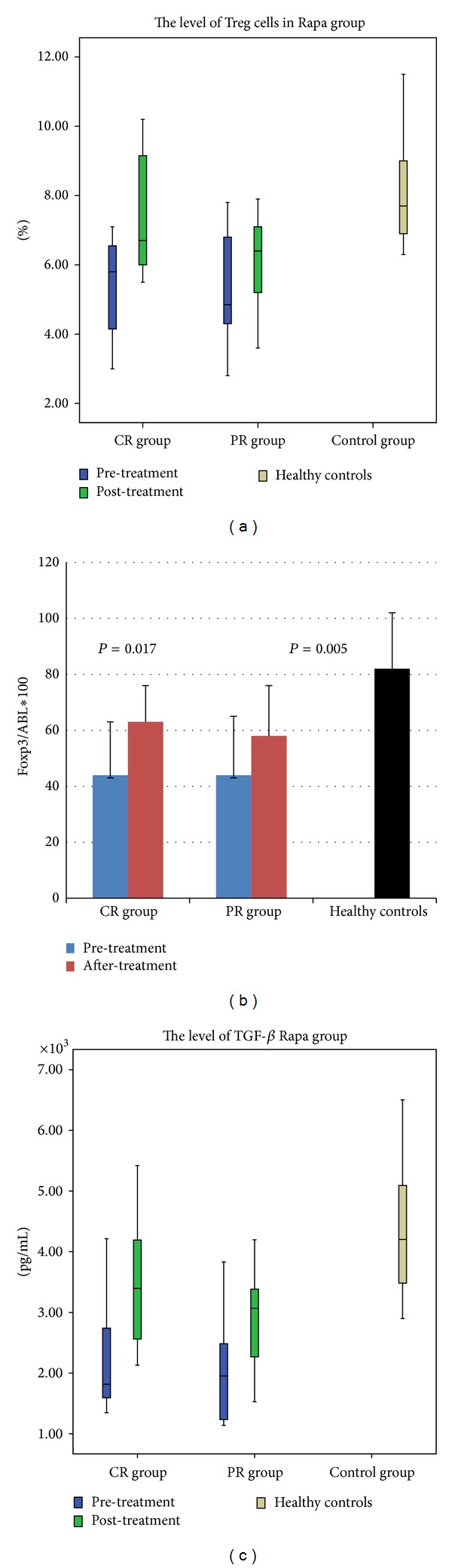
Immunologic assessment before and after the rapamycin or cyclosporine treatment. (a) The Treg cells levels by the flow cytometry. Patients with CR and PR in the experimental group experienced a marked upregulation (CR group: 5.35 ± 1.49% versus 7.55 ± 1.74%, *P* = 0.013; PR group: 5.17 ± 1.65% versus 6.12 ± 1.43%, *P* = 0.015); (b) the expression of Foxp3 mRNA by quantitative RT-PCR assay. It was consistent with the increase in Treg cells level (CR group: 44 ± 19 versus 63 ± 13, *P* = 0.017; PR group: 44 ± 21 versus 58 ± 18, *P* = 0.005); (c) the plasma levels of TGF-*β* by the enzyme-linked immunosorbent assay. A significant increase in the level of TGF-*β* was observed after the rapamycin treatment (CR group: 2.38 ± 1.0 ng/mL versus 3.57 ± 1.1 ng/mL, *P* = 0.028; PR group: 2.04 ± 0.92 ng/mL versus 2.90 ± 0.8 ng/mL, *P* = 0.001).

The pretreatment baseline levels of Treg cells were significantly lower in the two groups than those in the healthy controls (*P* < 0.001), and there was no difference between the two groups before the treatment (the rapamycin group versus the cyclosporine A group: 5.29 ± 1.52% versus 5.74 ± 1.42%, *P* = 0.155). However, following the rapamycin administration, patients with OR experienced a marked upregulation in the Treg cells level (*P* = 0.002) and the differences were statistically significant between the CR and PR groups (CR group: *P* = 0.013; PR group: *P* = 0.015) (Figure 1(a)). At the same time, the expression of Foxp3 mRNA was consistent with the increase in Treg cells level (CR group: *P* = 0.017; PR group: *P* = 0.005) (Figure 1(b)), while the increase in both the Treg cells level and the Foxp3 mRNA expression observed in the cyclosporine A group was minimal (Table 2).

#### 3.1.3. Cytokines

The TGF-*β* and IL-10 levels were low in both groups before the treatment. In the rapamycin group, a significant increase in the level of TGF-*β* was observed in OR patients after the treatment (OR group: *P* = 0.002; CR group: *P* = 0.028; PR group: *P* = 0.001) (Figure 1(c)), and a strong correlation was found between the rate of CD4^+^ Treg cells and TGF-*β* (*r* = 0.652, *P* = 0.001). There were no evident differences in the level of IL-2, IL-4, and IL-10 in both groups.

#### 3.1.4. Suppressive Capacity of the Treg Cells

**Figure 2:**
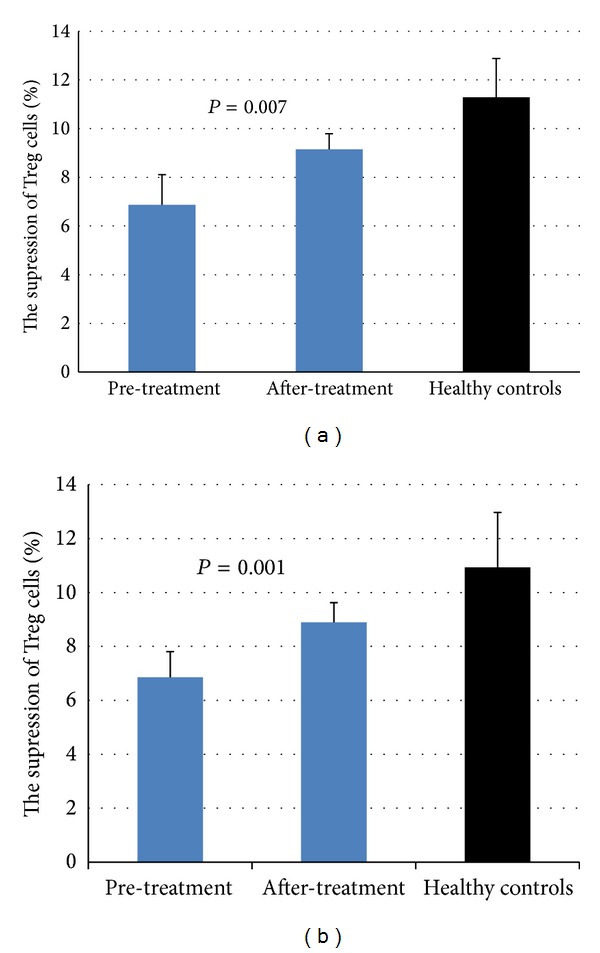
The suppressive activity of Treg cells before and after the treatment in the experimental group; (a) the suppressive ability for CD4^+^ T cells; (b) the suppressive ability for CD8^+^ T cells. The pretreatment baseline levels of Treg cells suppression was significantly lower for CD4^+^ cells and CD8^+^ cells than those in the healthy controls (6.87 ± 1.24% versus 11.28 ± 1.60%, *P* < 0.001; 6.86 ± 0.95% versus 10.93 ± 2.04%, *P* < 0.005). Following the rapamycin treatment, the suppression for CD4^+^ cells (9.15 ± 0.64%, *P* = 0.007) and CD8^+^ cells (8.89 ± 0.73%, *P* = 0.001) was stronger than that before the treatment.

**Figure 3:**
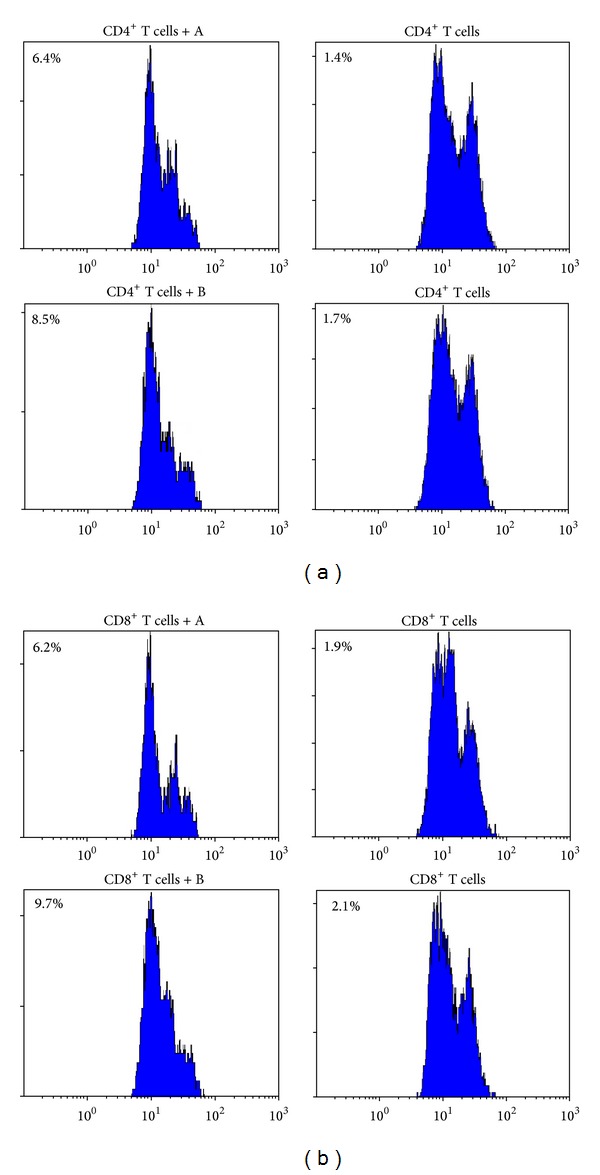
FCM analysis of suppressive Treg cells in vitro; (a) the suppression for CD4^+^ T cells by FCM analysis; (b) the suppression for CD8^+^ T cells by FCM analysis. The Treg cells were isolated from patient 5 (“A” means the Treg cells from the patient who was not given the rapamycin treatment; “B” means the Treg cells from the same patient who obtained CR), and their freshly purified autologous effector CD4^+^ T or CD8^+^ T cells were stimulated with anti-CD3 and anti-CD28. They were cocultured in culture medium. The cell division was assessed at day 5 after stimulation by FACS analysis of CFSE dilution. Suppression of responder cell proliferation is indicated as %.

No difference was seen between the rapamycin group and the cyclosporine group before the treatment (the suppression for CD4^+^ T cells: 6.87 ± 1.24 versus 6.72 ± 1.49, *P* = 0.669; the suppression for CD8^+^ T cells: 6.86 ± 0.95 versus 7.2 ± 0.88, *P* = 0.458). However, after the rapamycin administration, the suppression for both CD4^+^ cells (9.15 ± 0.64, *P* = 0.007) and CD8^+^ cells (8.89 ± 0.73, *P* = 0.001) was stronger than that before the treatment (Figure 2).  Figure 3 displayed the difference of functional Treg cells (from patient 5) between before and after the administration.

### 3.2. Follow-Up Phase

#### 3.2.1. Safety and Efficacy

No patients suffered the opportunistic or other severe infectious complication. In addition, there was no evidence of late hematological or extrahematological toxicity and none of the patients developed malignancies since the treatments cessation (the mean observation time: 18 months; range: 16–23 months). Eight patients were hospitalized because of the platelet decrease and bleeding (3 from the rapamycin group; 5 from the cyclosporine group). Three patients underwent splenectomy (2 from the rapamycin group, 1 from cyclosporine group).

The SR rate in the rapamycin group was 68% (in the CR group: 12/15 (80%); in the PR group: 5/10 (50%); and 39% in the cyclosporine group (in the CR group: 10/17 (59%); in the PR group: 1/11 (9%)).  Table 3 compared the prognostic factors between the SR group and the non-SR group. Six patients (24%) in the rapamycin group and 11 patients (39%) in the cyclosporine group relapsed after stopping the treatments.

#### 3.2.2. Immunological Assessment

The levels of Treg cells at baseline were similar between patients with SR and patients with non-SR (in the rapamycin group sustained responders versus nonsustained responders, *P* = 0.624; in the cyclosporine A group sustained responder versus nonsustained responders, *P* = 0.562; in the rapamycin group sustained responders versus in the cyclosporine A group sustained responders, *P* = 0.296; in the rapamycin group nonsustained responders versus in the cyclosporine A group nonsustained responders, *P* = 0.985). However, it was significantly increased in the SR group (the pretreatment level versus the posttreatment level: 5.44 ± 1.64 versus 6.60 ± 1.34, *P* < 0.005); in particular, the percentage of Treg cells in the rapamycin group was most elevated (*P* < 0.005), and maintained stable during the follow-up phases (Table 4).

## 4. Discussion

ITP patients had an associated mortality from severe bleeding and drugs toxicities, while the options for the management were limited. Steroid combined with intravenous immunoglobulin and/or anti-Rh (D) immunoglobulins is the standard treatment regimen for ITP; unfortunately, the effects of these treatments are transient; Rituximab or thrombopoietic agents are significantly effective, but they are less cost-effective than the standard administration; the splenectomy may help improve the platelet counts, while most of the patients are reluctant to have an operation. Therefore, an effective and economical treatment was poorly needed.

It is the first time that the effects of rapamycin on patients with ITP have been investigated. In this study, we provided one promising approach: Steroid is the first-line treatment regimen for ITP, which can improve promptly the platelet count; rapamycin is able to inhibit the self-reactive T cells but selectively promote the Treg cells. In this condition, patients could obtain a rapid and persistent response.

Rapamycin and cyclosporine are both calcineurin pathway inhibitors, which selectively inhibit antigen-induced activation of CD4^+^ lymphocytes. Many clinical trials have displayed that cyclosporine has been associated with a platelet count response in 44% to 75% of ITP patients [30–33]. In our study, both groups had the similar OR (58% versus 62%). However, through the comparison of Treg cells, patients obtaining OR in the rapamycin group experienced a significant upregulation and a strong correlation was found between the percentage of Treg cells and TGF-beta. Similarly, the previous studies have confirmed that only the local inflammatory cytokine milieu was controlled; the Treg cells would exert their suppressive function on established autoimmune disease [34, 35]. To clarify the possible mechanism, we examined the suppressive activity of Treg cells before and after the treatment and found that rapamycin could enhance the suppression of Treg cells in vitro. These findings suggest that the rapamycin treatment may play a role in improving the function of Treg cells, either directly or indirectly by enhancing the release of TGF-beta.

Yun et al. investigated the changes of Treg cells in adult ITP patients before and after treatment with high dose dexamethasone and found that the immunosuppressive therapy of glucocorticoids could cause the short-time increase of Treg cells. Recently, the treatments with Rituximab or thrombopoietin receptor agonists can also improve the Treg cells level, while longer-term studies are still required to validate the clinical significance of the elevation of Treg cells observed in patients. In our study, we found that the level of Treg cells in SR patients in the rapamycin group was increased and maintained stable. In addition, during the follow-up phase, the SR rate was greater in the rapamycin group than in the cyclosporine group. Comparing the prognostic factors between the sustained responsive group and the nonresponsive group, the outcome does not appear to be affected by age, sex, previous treatments, baseline platelet count, and baseline megakaryocyte (MEG) counts in bone marrow slides. Therefore, the rapamycin treatment provides a novel method with long-lasting response and low relapsed rate, which can increase and maintain the level of functional Treg cells, to treat ITP.

However, the follow-up duration in our study is not long enough, so the evaluation of response will continue; 6 patients in the rapamycin group relapsed off treatment and 4 out of them showed the same response when they were given the rapamycin treatment again, which may suggest that there were platelet-specific Treg cells in the peripheral blood.

In conclusion, results from this study suggest that rapamycin combined with low dose prednisone could increase the platelet count significantly and promote the proliferation of functional Treg cells. For ITP patients who failed to respond to other treatments, the novelty therapy may be attractive because of its low toxicity and lasting SR. Further studies will be needed to investigate the characterization of platelet-specific Treg cells and which ITP patients would be best suitable for the rapamycin administration.

## Figures and Tables

**Table 1:** Patient's clinical and laboratory characteristics.

Clinical and laboratory characteristics	Rapa group	CsA group	*P*
Patients number	43	45	
Females/males	29/14	32/13	0.713
Mean age, years	34 (13–65)	36 (14–58)	0.456
Mean duration, years	3 (2–6)	3 (2–6)	0.420
Mean bleeding grade (WHO Bleeding Scale)	1 (0–2)	1 (0–2)	0.377
Mean baseline platelet count, ×10^9^/L	22.25 (11–32)	21.82 (11–35)	0.747
Mean MEG count in bone marrow slide	105 (34–231)	100 (34–241)	0.710
Platetle antibody, yes/no	28/15	28/17	0.781
Mean days since the last treatment	63 (10–365)	57 (10–365)	0.664
Mean numbers of previous treatments	3 (2–6)	3 (2–5)	0.106
Previous treatments number	43	45	
Steroids	43	45	
Intravenous immune globulin	17	21	
Danazol	16	19	
Azathioprine	10	14	
Vincristine	2	1	
Rituximab	1	0	

The previous treatments included Steroids, intravenous immune globulin, Rituximab, Danazol, Azathioprine, and Vincristine; the determination of platelet antibody included glycoproteins IIb/IIIa/Ib/IX.

MEG: megakaryocyte; Rapa: rapamycin; CsA: cyclosporine A.

**Table 2:** The level of Treg cells before the treatment and after the treatment.

Group	The rapamycin group		The cyclosporine A group		HC
	Pre-t	Post-t	Pre-t	Post-t	
OR	5.28 ± 1.52	6.98 ± 1.7^*※*^	5.63 ± 1.55	6.17 ± 1.65	8.15 ± 1.55
NR	5.30 ± 1.55	6.1 ± 2.02	5.92 ± 1.19	6.15 ± 1.79	

Pre-t: before the treatment, Post-t: after the treatment.

HC: healthy controls.

^*※*^ *P* < 0.01, comparing the Treg cells levels before the treatment to that after the treatment.

**Table 3:** Comparison of prognostic factors between sustained responsive group and nonsustained responsive group.

Clinical and laboratoristic prognostic factors	The SR group	The non-SR group	*P*
Patients' number	28	25	
Males/females	12/16	6/19	0.154
Mean age, years	32 (14–58)	36 (14–65)	0.302
Mean duration, years	3.07 (2–6)	2.88 (2–5)	0.504
Mean baseline Plt, ×10^9^/L	22.96 (11–31)	21.96 (11–35)	0.581
Mean MEG count in bone marrow slide	107 (34–213)	91 (34–147)	0.088
Mean numbers of previous treatments	3 (2–4)	3 (2–5)	0.584

The SR/non-SR group included patients with SR/non-SR in both groups; MEG: megakaryocyte; Plt: platelet.

**Table 4:** The comparison of the Treg cells level between patients with SR and with non-SR.

		Before the treatment (%)	During the follow-up phase (%)
The rapamycin group			
SR	17	5.17 ± 1.50	6.94 ± 1.40^*※*^
Non-SR	8	5.5 ± 1.65	6.01 ± 1.22

The cyclosporine A group			
SR	11	5.84 ± 1.82	6.07 ± 1.14
Non-SR	17	5.49 ± 1.39	5.11 ± 1.01

^*※*^ *P* < 0.005, comparing the Treg cells level before the treatment to that during the follow-up phase in the rapamycin group.
